# Effects of inpatient treatment of adolescents with anorexia nervosa are associated with body representation: a monocentric pilot study

**DOI:** 10.1038/s41598-025-13222-w

**Published:** 2025-08-01

**Authors:** Tasuku Kitajima, Toshihiro Kawase, Satoshi Shimada, Takeshi Inoue, Ryoko Otani, Toshimitsu Takahashi, Soichiro Fujiki, Kensaku Nomoto, Kenji Kansaku, Ryoichi Sakuta

**Affiliations:** 1 Child Development and Psychosomatic Medicine Center, Saitama Medical Center, Dokkyo Medical University, Saitama, Japan; 2 Department of Pediatric Psychosomatic Medicine, Nagasaki Prefectural Center of Medicine and Welfare for Children, Nagasaki, Japan; 3 Department of Information and Communication Engineering, School of Engineering, Tokyo Denki University, Tokyo, Japan; 4 Department of Physiology, Dokkyo Medical University School of Medicine, Tochigi, Japan

**Keywords:** Anorexia nervosa, Body representation, Inpatient treatment, Rubber Hand Illusion, Robot Hand Illusion, Human behaviour, Psychiatric disorders, Psychology, Diseases

## Abstract

This monocentric pilot study investigates the relationship between nutritional treatment and body representation distortion in adolescents with Anorexia Nervosa (AN) using the Rubber Hand Illusion (RHI) task. A total of 28 adolescents participated, including 14 patients with restrictive AN and 14 healthy controls. The RHI and Robot Hand Illusion (RoHI) tasks were conducted, with assessments taken at admission and discharge for the AN group. Sense of Ownership (SO) and Sense of Agency (SA) rated via 7-item Likert; proprioceptive drift was millimetre shift in perceived hand position after stimulation vs baseline under congruent (sync/in-phase) or incongruent (async/out-of-phase) feedback. Results showed that all groups exhibited higher SO scores in the synchronous condition compared to the asynchronous condition in the RHI task. In the RoHI task, AN patients at admission displayed significantly higher SA scores in the in-phase than in the out-of-phase condition. Notably, SO scores in the RHI task at discharge were positively correlated with weight recovery one month post-discharge (*ρ* = 0.59). These findings suggest that inpatient treatment influences body representation in adolescents with AN and that changes in body perception may serve as an indicator of treatment effectiveness.

## Introduction

Anorexia nervosa (AN) is a life-threatening psychiatric disorder characterized by an overvaluation of body shape and weight. As several studies and meta-analyses report body image deficits in individuals with AN^1–3^, individuals with AN experience change in the process of recognizing their bodily self, which may contribute to their symptoms^4,5^. In other words, people with AN may not only intentionally seek to lose weight and exercise, but they may also have disturbances in their bodily self-perception and struggle to recognise their bodies accurately, construct a body image, or maintain control over their bodies. Understanding these changes in the perception of body representation is an important insight that may lead to new approaches to body image disturbance and neurophysiological treatments.

Two senses play a fundamental role in bodily self-awareness: sense of ownership (SO) and sense of agency (SA). SO is the feeling of mineness—the intuition that “this is my hand” or “I am the one having this sensation”. SA is the phenomenal experience of initiating and controlling an action, the authorship we invoke when stating “I am controlling this car” or “I must have pressed this button”^6^. These senses are critical for representing our body^7^, and for constructing and updating the body model of the self^8^. The Rubber Hand Illusion (RHI)^9^ is widely used to study SO, while actively moving a rubber hand simultaneously elicits SA to the rubber hand^10,11^. Our group developed an active RHI with a robotic hand, the Robot Hand Illusion (RoHI), and reported that this paradigm allows us to experience SA to the robotic arm^12^. These tasks are well suited for isolating sensory-driven and motor-driven components of embodiment, allowing researchers to examine SO and SA as partially dissociable constructs.

RHI studies have shown altered body representation in individuals with eating disorders, who are more prone to illusion experiences (i.e., stronger RHI/RoHI effects) and exhibit correlations with eating disorder severity^13–15^. Patients who regain weight and abstain from eating disorder behaviors for over a year show intermediate levels of RHI susceptibility between acute patients and healthy controls^13^. By contrast, adding a motor component stabilizes SO, resulting in responses comparable to healthy controls^15^. These findings suggest that classical RHI may reveal vulnerability, while motor-induced conditions reflect SA-driven stability.

Unresolved issues in prior studies include mixed eating disorder subtypes, which may obscure differences in body representation. For example, binge-eating disorder shows unclear associations with body image disturbances^16^. Additionally, prior studies focused on adults with long-term illness, possibly introducing secondary effects due to chronic undernutrition, as indicated by other studies^17–19^. Research targeting adolescents shortly after onset can minimize such confounding factors. Furthermore, longitudinal studies are limited, despite evidence that body representation and brain function in AN can change with weight gain during short-term re-nutritional therapy^20^.

The relationship between RHI effect levels and eating disorder severity has often been interpreted negatively as a sign of vulnerability^14^. However, severity does not always predict prognosis, emphasizing the need to examine the association between RHI illusion levels and treatment outcomes.

This monocentric pilot study conducted in Japan aimed to assess RHI and RoHI tasks in Japanese adolescents with AN shortly after onset, both before and after inpatient nutritional rehabilitation. To date, studies investigating the relationship between RHI results and longitudinal clinical outcomes in people with eating disorders are extremely limited. We hypothesized that flexibility in body representation influences the effectiveness and prognosis of inpatient nutritional rehabilitation in adolescents with anorexia nervosa; therefore, we undertook this preliminary study to explore the potential implications of body representation changes for treatment outcomes, providing a basis for future large-scale research.

## Methods

### Participants

Participants in the AN group, aged 11–18 years, were recruited from patients admitted to the inpatient nutritional rehabilitation program at the Child Development and Psychosomatic Medicine Centre, Saitama Medical Centre, Dokkyo Medical University. Both the participants and their parents gave informed consent to participate in the study. They were diagnosed according to the DSM-5 by board-certified child mental health medical specialists. All participants in the AN group were monitored for more than 1 year, and their diagnoses, all restrictive type AN, were confirmed. One of the 14 participants withdrew from the inpatient program and another refused to participate in the second experiment.

The healthy control (HC) group consisted of 13- to 15-year-old volunteers who were recruited at a junior high school in Saitama Prefecture, Japan. The control participants were confirmed to have no history of mental illness, including eating disorders, by self-report and the Mini-International Neuropsychiatric Interview for children/adolescents^21^. Their height and weight were measured when they participated in the experiment and this confirmed that they had a body weight (BW) to ideal body weight (IBW) ratio based on height and age (%IBW) of at least 80%. IBW was determined using the formula for sex, age, and height published by the Japanese Society for Paediatric Endocrinology^22^.

This study was approved by the Ethics Review Committee of Saitama Medical Centre of Dokkyo Medical University (No. 2045) and was conducted with the participants’ consent in accordance with the Declaration of Helsinki. Written informed consent was obtained from the participants’ parents, and informed assent was obtained from the adolescent participants themselves after providing them with an age-appropriate explanation of the study.

### Inpatient nutrition rehabilitation program

The inpatient program at the Child Development and Psychosomatic Medicine Centre is a standard inpatient treatment program in Japan, as outlined by the guidelines of the Working Group on Eating Disorders, Japanese Society of Psychosomatic Pediatrics^23^. The goal is to progressively increase the nutritional intake for severe undernutrition due to AN, aiming to achieve a weight regain of at least 75–80% relative to the IBW. At admission, all patients are started on oral or tube feeding at 20–30 kcal/day (starting at a lower dose for severe undernutrition) with a continuous peripheral infusion containing maintenance fluids with added phosphorus. Once the patient can eat served dishes completely, the calories are increased every 2–3 days, and the continuous infusion is tapered accordingly. Once the intake reaches 1400–1600 kcal/day, the infusion is withdrawn, and the patient is eventually encouraged to eat more than 2400–3000 kcal/day. All adolescents with AN in this study were admitted and treated following this protocol. They were followed-up every 1–2 weeks for the first month after discharge.

### Schedule of the experiment

The AN group participated in two experiments, one at the beginning of hospitalization and the other just before discharge. The first experiment was conducted when the infusion was stopped (T1; mean 20.2 ± 16.5 days from admission), because all patients were on continuous peripheral infusion at the time of admission. The HC group was initially scheduled to undergo the second experiment approximately 3 months later, at the same time as the AN group. However, due to the COVID-19 pandemic, only the first experiment was conducted in the HC group.

### Procedure

#### Rubber Hand Illusion task (RHI)

**Fig. 1:**
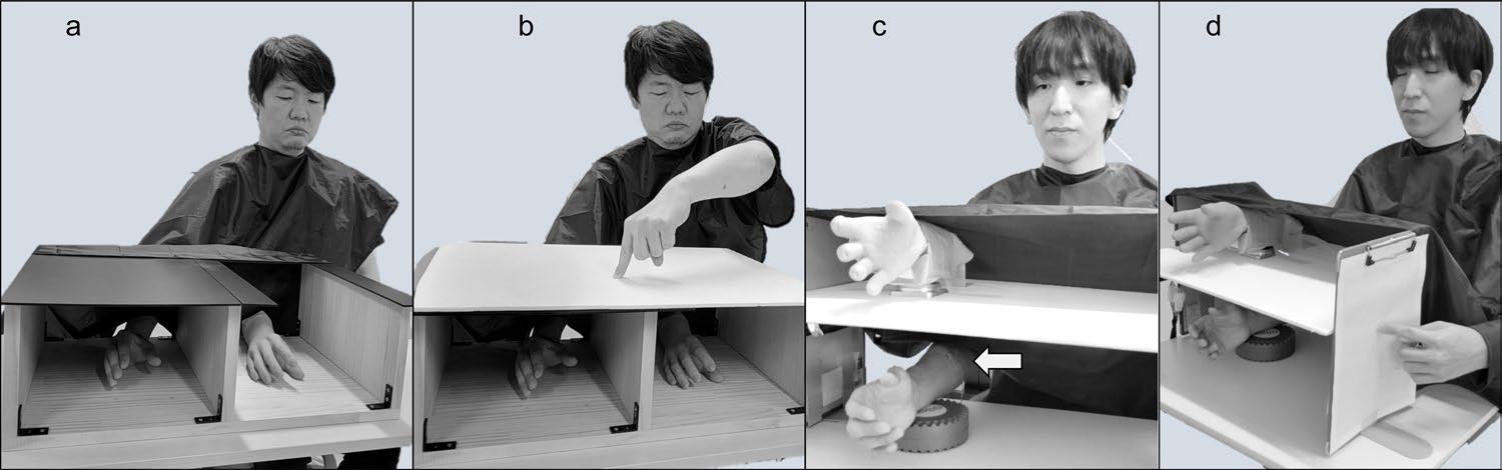
The Rubber (**a**,**b**) and Robot (**c**,**d**) Hand Illusion tasks. In the Rubber Hand Illusion task, participants place their own hand in the black box and gaze at the rubber hand (**a**). Before and after the brush stroke, the participant points with the opposite hand at the fingertip position of the index finger of his/her right hand and the horizontal difference before and after the task is calculated (**b**: proprioceptive drift). In the robot hand illusion task, the participant places his/her own hand under the rubber hand and activates the robotic hand while gazing at the rubber hand (**c**). The joint position of the robotic arm was continuously controlled by the participant’s muscle activity via two electrodes (arrow in c) that captured electromyographic signals from the flexor carpi radialis and extensor carpi ulnaris muscles. Before and after moving the robotic arm, the participant points with the opposite hand at the fingertip position of the index finger of his/her right hand and the vertical difference before and after the task is calculated (**d**: proprioceptive drift).

Participants were seated at a table opposite the experimenter and instructed to place their hand in a box. A life-size right-hand prosthetic glove (model 8S11N; Otto Bock, Duderstadt, Germany) was placed in the box on the right side of the participant’s midline, with the participant’s own right hand shielded from view and only the prosthetic glove visible. The distance between the participant’s own hand and the rubber hand was approximately 20 cm. The experimenter sat opposite the participant and stroked both the real hand and prosthetic glove with a brush at approximately 1 Hz for 120 s. While stroking, the participant was asked to gaze at the rubber hand (Fig. 1a). The brush strokes were performed twice under each condition (a synchronous condition, where brush strokes were applied simultaneously to both hands in time and location, and an asynchronous condition, where strokes were mismatched in time and location) between the participant’s hand and rubber hand. The synchronous condition was designed to induce the SO over the rubber hand, while the asynchronous condition served as a control to suppress the illusion. The asynchronous strokes differed from each other by 180° and stroked different locations on the hand. The questionnaire measuring the SO toward the rubber hand was completed after the strokes and the proprioceptive drift was measured before and after the strokes (Fig. 1b).

#### Robot Hand Illusion task (RoHI)

We built a one-degree-of-freedom (wrist flexion and extension) robotic arm with proportional myoelectric control^24^ that consisted of a prosthetic glove (model 8S11N; Otto Bock, Duderstadt, Germany) resembling a real hand in size, shape, and appearance and an actuator (model FHA-11 C; Harmonic Drive LLC, Peabody, MA). The joint position of the robotic arm was continuously controlled by the participant’s muscle activity via two electrodes that captured electromyography (EMG) signals from the flexor carpi radialis and extensor carpi ulnaris muscles. Each signal was captured at 1000 Hz by a wireless EMG recorder (AvatarEEG; Electrical Geodesics, Eugene, OR, USA) and sent to a personal computer in real time via a Bluetooth connection. To extract information about muscle activity from the EMG signals, the signals were high-pass filtered (4th order Butterworth, cut-off frequency 60 Hz), digitally rectified, and low-pass filtered (2nd order Butterworth, cut-off frequency 3 Hz)^25^. The robotic arm was calibrated using MATLAB (MathWorks, Natick, MA, USA). During the calibration phase, the participants performed several repetitions of alternating wrist flexion and extension, synchronised with the motion of the robotic arm. A computer-based algorithm used these EMG signals to identify the neural network parameters for estimating the participant’s wrist angle^26^. After calibration, flexion, and extension of the participant’s wrist resulted in simultaneous matched movements of the robotic arm.

Using EMG of the forearm flexor and extensor muscles, the participant drove the robotic arm for a fixed period (Fig. 1c). The participant could see only the robotic hand, as the right hand on the same side as the robotic arm was shielded from view. The robotic arm was covered with the same rubber hand that was used in the RHI, giving it the appearance of a real hand. In the in-phase condition, the robotic hand moved synchronously with the wrist movement estimated from the participant’s EMG signals, while in the out-of-phase condition, the movement was reversed relative to the estimated movement. The in-phase condition was intended to enhance the SA and SO, whereas the out-of-phase condition disrupted this sensorimotor congruence. In the RoHI, as in the RHI, the SA and SO toward the rubber hand were measured using questionnaires after moving the robotic arm, in addition to measuring the proprioceptive drift (Fig. 1d).

### Proprioceptive drift

Before the stroke, the participant was told to point to the position of the shielded hand using the index finger of the non-shielded hand. The same instruction was given after the stroke and the shift in pointing position before and after the stroke was calculated (Fig. 1b,d). The degree to which the sense of body affiliation shifted from self to the rubber hand was correlated with the positional drift; horizontal proprioceptive drift was measured in RHI, and vertical proprioceptive drift was measured in RoHI.

### Measurements of embodiment

Embodiment was measured immediately after each experimental condition. The robot hand illusion consisted of the same content as the RHI task, plus three agency statements and three control statements. For each statement, the respondents responded on a 7-point Likert scale (1 = strongly disagree, 7 = strongly agree)^27^. The scores of the SO items—“I felt as if I was looking at my own hand”, “I felt as if the rubber hand was part of my body”, and “I felt as if the rubber hand was my hand”—were summed to yield the SO rating. Likewise, the scores of the SA items—“I felt as if I could cause movements of the rubber hand”, “I felt as if I could control movements of the rubber hand”, and “The rubber hand was obeying my will, and I could make it move just as I wanted”—were summed to yield the SA rating.

### Psychological measurements

#### Children’s Eating Attitudes Test (Japanese version)

The Children’s Eating Attitudes Test is a 26-item, self-administered test that assesses body image, food obsessions/preoccupations, and dieting on a 5-point Likert scale (higher scores = greater disordered-eating attitudes)^28^. The Japanese version has been validated for reliability and validity^29^.

#### Eating Disorder Examination Questionnaire (Japanese version)

This is a self-administered 7-point Likert scale test used to screen for eating disorders and assess their severity^30^. It contains 22 items that identify eating disorder symptoms over the past 28 days (higher scores = more severe eating-disorder symptoms). The Japanese version has been validated for reliability and validity^31^.

#### Spence Child Anxiety Scale (Japanese version)

This is a self-administered 4-point Likert scale consisting of 38 items that assess anxiety in children on six subscales (separation anxiety disorder, social anxiety disorder, obsessive–compulsive disorder, panic disorder, generalised anxiety disorder, and traumatic fear) (higher scores = higher anxiety levels). The Japanese version has been validated for reliability and validity^32,33^.

#### The Birleson Depression Scale for Children (Japanese version)

This is a self-rated 3-point Likert scale for depression in children, consisting of 18 questions (higher scores = greater depressive symptomatology)^34^. The reliability and validity of the Japanese version have been verified^35^.

### Statistical methods

The statistical analyses were conducted using MATLAB ver. 24.1 using Statistics and Machine Learning Toolbox. The normality of the dataset was examined using the Shapiro–Wilk test, and between-group comparisons for background and clinical data were performed using one-way ANOVA (normal) and the Kruskal–Wallis test (non-normal). Due to insufficient normality in the RHI and RoHI datasets, nonparametric tests were used. The Wilcoxon signed-rank test was used to compare synchronous (in-phase) versus asynchronous (out-of-phase) conditions for AN (T1), AN (T2), and HC, calculating the effect size r. Two-tailed Spearman’s rank correlation coefficient was employed to examine the correlation between change in %IBW one month after discharge and SO score on RHI, and SA score on RoHI at T2. For Spearman’s *ρ*, CIs were computed using bias-corrected and accelerated (BCa) bootstrapping with 10,000 resamples. For correlation and group-level analyses, cases with missing data at a given time point were excluded list-wise, while data from the same participant at other time points were retained if complete. No data were excluded as outliers. Because all group comparisons and correlation analyses were conducted using rank-based non-parametric methods, the influence of extreme values was inherently minimized. The figures were generated using Python ver. 3.8.10 (Python Software Foundation, Wilmington, DE, USA) with Pandas, NumPy, Matplotlib, Seaborn, and SciPy libraries.

## Results

### Participants’ clinical backgrounds

Twenty-eight adolescents participated in the study: 14 with AN (all restrictive type) and 14 HC. The mean duration of the disease for the adolescents with AN at time T1 was 7 months. After the initial RHI at admission, data from both the RHI and RoHI at discharge were available for 12 patients. Table 1 summarizes the participants’ background, clinical data, and psychological scores. At T1, the mean age of AN was 1 year younger than that of HC, but there were no significant differences among the AN (T1), AN (T2), and HC groups. There were no significant differences in mean height, while weight, BW, and BMI were all significantly lower in the AN group. Both depression and eating disorder pathology scales were higher in AN (T1) than in HC. AN (T2) had lower scores on average than AN (T1) for eating disorder pathology, anxiety, and depression scales, but the differences were not significant.

**Table 1:** Clinical characteristics of the participants.

	AN group (T1) n = 12	AN group (T2) n = 12	HC group n = 14	*P*	Post-hoc analysis
Age (month)	160.2 (16.6)	162.8 (17.7)	172.1 (5.1)	0.080	
Illness duration (month)	7.0 (4.2)				
Height (cm)	153.8 (6.9)	154.0 (7.2)	157.4 (5.5)	0.284	
Weight (kg)	29.9 (5.4)	37.7 (5.2)	49.2 (5.4)	< 0.001	T1 < T2, T1 < HC, T2 < HC
%IBW	64.1 (4.7)	81.0 (1.6)	98.3 (8.7)	< 0.001	T1 < T2, T1 < HC, T2 < HC
BMI	13.1 (10.6–14.1)	15.8 (14.2–16.9)	19.8 (16.8–23.6)	< 0.001	T1 < T2, T1 < HC, T2 < HC
BMI-SDS	− 4.48 (0.98)	− 1.82 (0.23)	− 0.17 (0.66)	< 0.001	T1 < T2, T1 < HC, T2 < HC
DSRS-C	17.7 (8.4)	12.2 (6.5)	7.8 (5.6)	0.003	T1 > HC
SCAS	48 (8–87)	15.5 (2–73)	17 (3–55)	0.101	
ChEAT26	38 (7–53)	14 (4–59)	8 (2–30)	< 0.001	T1 > HC
EDE-Q-J	2.68 (1.60)	1.98 (1.79)	1.06 (0.81)	0.02	T1 > HC

Variables with a normal distribution are presented as mean (standard deviation), while non-normally distributed variables are presented as median (minimum–maximum).

IBW, ideal body weight; BMI, body mass index; SDS, standard deviation score; DSRS-C, Birleson Depression Self-Rating Scale for children; SCAS, Spence Child Anxiety Scale; ChEAT26, Children’s Eating Attitudes Test; EDE-Q-J, Eating Disorder Examination Questionnaire Japanese version; AN, anorexia nervosa; HC, healthy control.

*P* values indicate results of one-way ANOVA or Kruskal–Wallis test, depending on data distribution.

### Rubber Hand Illusion task

**Fig. 2:**
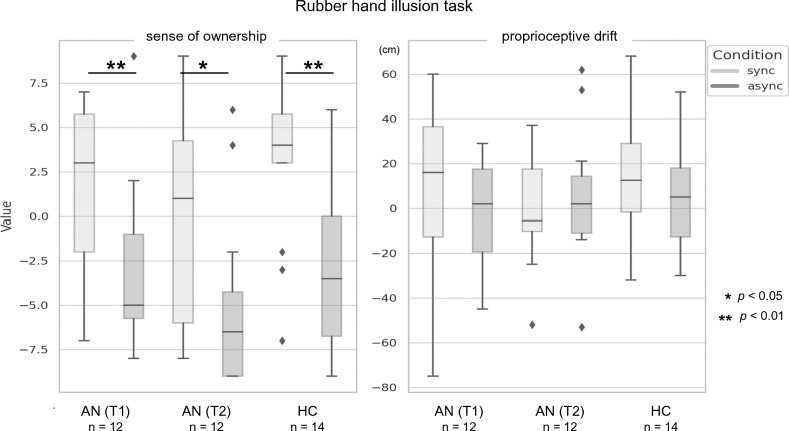
Results of the Rubber Hand Illusion task. In all groups—AN at T1, AN at T2, and HC—SO scores were significantly higher in the synchronous condition compared to the asynchronous condition. AN, anorexia nervosa; HC, healthy control.

In the RHI results, the SO scores were significantly higher in the synchronous condition for both the AN (T1) and (T2) groups and the HC (Fig. 2) (T1: *p* < 0.01, r = 0.74; T2: *p* = 0.01, r = 0.67; HC group: *p* < 0.01, r = 0.79). By contrast, the proprioceptive drift did not differ significantly in the synchronous vs. asynchronous conditions in either AN group (T1, T2) or HC (T1: *p* = 0.26, r = 0.31; T2: *p* = 0.30, r = 0.28; HC: *p* = 0.15, r = 0.38).

### Robot Hand Illusion task

**Fig. 3:**
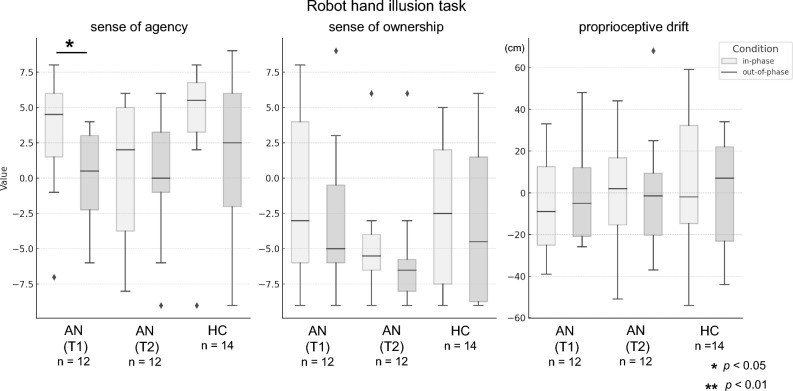
Results of the Robot Hand Illusion task. In the AN group at T1, the SA score was significantly higher in the in-phase condition compared to the out-of-phase condition. In contrast, no significant difference in SA scores was observed between conditions in either the AN group at T2 or the HC group. AN, anorexia nervosa; HC, healthy control.

In the AN group, 12 each T1 and T2 RoHI trial datasets were obtained by excluding data from RoHI results with a coefficient of determination (R^2^) of the neural network of the robotic hand of less than 0.1 (two trials in T1). In the RoHI, SA scores in the AN (T1) group were significantly higher in the in-phase condition than in the out-of-phase condition (*p* = 0.01, r = 0.70), whereas no significant difference was observed in the AN (T2) group (*p* = 0.95, r = 0.02) (Fig. 3). There was a slight but nonsignificant difference in the HC group (*p* = 0.06, r = 0.48), which may have been attributable to the small group size. There were no significant differences between the in-phase and out-of-phase conditions for SO and proprioceptive drift in either case.

### Correlation between the sense of ownership on RHI task and %IBW change after the discharge

**Fig. 4:**
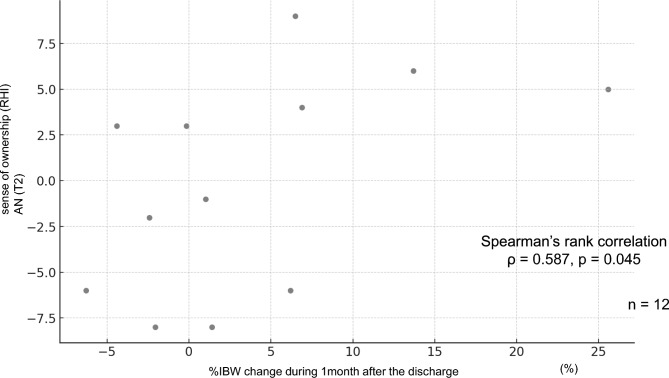
Correlation between the sense of ownership score at T2 and %IBW change 1 month after discharge. In the AN group, SO scores in the synchronous condition of the RHI task at T2 (i.e., prior to discharge) were positively correlated with the change in %IBW one month after discharge (*ρ* = 0.587, *p* = 0.045). RHI, Rubber Hand Illusion; AN, anorexia nervosa; IBW, ideal body weight.

To investigate the relationship between the RHI task results and short-term prognosis, the correlation between the SO scores in the RHI task (synchronous condition) at discharge (T2) and the change in %IBW 1-month post-discharge in the AN group was examined (Fig. 4). The SO scores in the synchronous condition of the RHI task were positively correlated with %IBW change (*ρ* = 0.59, *p* = 0.04; 95% BCa bootstrap CI  − 0.01 to 0.84).

### Correlation between the sense of agency on RoHI task and %IBW change after the discharge

The SA scores in the synchronous condition of the RoHI task (in-phase condition) were not correlated with %IBW change (*ρ* = 0.49, *p* = 0.10; 95% BCa bootstrap CI  − 0.19 to 0.89) (data not shown).

In an exploratory analysis, we also examined Spearman’s rank correlations between %IBW change after discharge and other embodiment-related measures, including SO, SA, and proprioceptive drift scores from the RHI and RoHI tasks at T1, T2, and their change scores (T2 minus T1). The full correlation matrix is provided in Supplementary Table S1. Aside from the significant correlation between %IBW change and SO in the synchronous condition of the RHI task at T2, no other statistically significant associations were observed.

## Discussion

- This study was the first longitudinal investigation of changes in bodily self-perception before and after short-term re-nutrition treatment using the RHI, as well as its relationship with subsequent short-term changes in weight. We also performed the RoHI for the first time in people with AN.
- We found a correlation between SO ratings in the synchronous condition of the RHI task at discharge (T2) and weight change one month post-discharge, suggesting that degree of RHI at discharge may reflect the treatment effect. Higher SO ratings in AN have previously been linked to vulnerability in body image^13,14^. In our pilot sample, however, we observed that higher SO scores coincided with a favourable short-term weight outcome. This raises the tentative possibility that elevated SO might index a greater malleability (i.e., plasticity) in body representation. Given the limited sample size and observational design, this interpretation remains speculative and should be tested in larger, longitudinal cohorts before any firm conclusion can be drawn. Previously, it was reported that the rubber hand in the RHI task is more likely to produce the RHI effect of visual similarity to one’s own hand^36^. The rubber hand used in this study was adult-sized, potentially causing adolescents with AN, particularly after weight recovery, to perceive it as “fat” and reject its identification as their own. This top-down suppression could reflect a protective mechanism against the RHI effect. This finding suggests that implicit acceptance of the “fat” rubber hand at discharge could be associated with better treatment outcomes. However, the average SO score was paradoxically higher at T1, when eating disorder pathology was more severe. This appears inconsistent with a purely top-down suppression mechanism, suggesting that other factors may contribute to the RHI experience in early-stage AN. The RoHI task, in contrast, showed greater resistance to the RoHI effect at discharge (T2) and did not correlate with changes in body weight after discharge, which may indicate a different mechanism.

In the RHI task, the SO values were higher in the synchronous condition than in the asynchronous condition for the AN group. By contrast, there were no significant differences in proprioceptive drift between the AN and HC groups. This result differs from previous reports. Note that the subjective ownership rating and proprioceptive drift measure different processes^7,37,38^. Rohde et al. suggested that changes in bodily possession are not always accompanied by proprioceptive shifts^37^, and Tosi et al. emphasized that these two measures reflect distinct processes, often confounded by small sample sizes^38^. While the association between these two measures was not the primary focus of the current study, the potential impact of a small sample size should not be disregarded.

Previous studies that performed the RHI on people with AN have reported a trend toward a greater RHI effect in the AN group compared to healthy controls^13,14^. Although those studies used ANCOVA for their analyses, we could not apply this due to the non-normality of the RHI and RoHI measurements. However, there was no obvious trend toward a greater RHI effect in the AN group than in the healthy controls, contradicting those previous studies. This suggests that enhanced susceptibility to the RHI in AN might be a “scar” symptom related to chronic undernutrition rather than an early disease trait. Sensory processing characteristics observed in AN align with this interpretation^19^. It remains essential to distinguish between trait symptoms present before onset, state symptoms during illness, and scar symptoms persisting after remission.

A key strength of this study is its focus on adolescents with AN in the early stages after onset, minimizing the influence of long-term disease effects and secondary symptoms caused by prolonged undernutrition. The use of both the RHI and RoHI tasks enabled a comprehensive evaluation of body representation, providing novel insights into the relationship between body representation and treatment outcomes. Furthermore, the inclusion of findings from a Japanese population addresses the scarcity of research on eating disorders in non-Western contexts, as highlighted by Marzola et al.^39^, contributing to a more global understanding of AN.

In the present study, healthy controls and patients with AN did not show a consistent increase in SA under the in-phase RoHI condition. Beyond the limited sample size, this finding may be partly explained by evidence that adolescence is characterized by a transient reduction in the implicit sense of agency^40^. Moreover, emerging data suggest that SA is specifically altered in AN^41^. Taken together, our results raise the possibility that SO and SA follow distinct recovery trajectories during inpatient treatment. Larger longitudinal studies with finer age stratification are required to clarify developmental fluctuations in SA and their relevance to treatment response.

However, several limitations should be noted. First, this monocentric pilot study included a small sample, which greatly limits statistical power. Based on our previous results using healthy adult participants^12^, the statistical power to detect the difference in the sense of ownership between the in-phase and out-of-phase conditions of the RoHI task in the HC group was *β* = 0.98 with the current sample size. However, assuming the effect size observed in the present study, the power was reduced to *β* = 0.39. As a result, the non-significant RoHI findings cannot be taken as evidence of no effect; rather, they remain inconclusive and require confirmation in larger cohorts. Future studies with adequate sample sizes, informed by the effect-size estimates obtained here, are needed to verify the reproducibility and generalisability of our results. Additionally, adolescence is a period of significant physical, emotional, and brain development, which may influence body representation. Ferracci and Brancucci reported that younger individuals tend to experience a stronger and more rapid onset of the RHI, which could have affected our results. In this study, the HC group was, on average, about one year younger than the AN (T1) group, potentially introducing age-related differences. However, no clear trend was observed between age and responsiveness to the RHI or RoHI in the HC group (results are shown in the Supplementary Fig. S2), suggesting that age did not have a significant impact on our findings. Third, this study was limited to adolescent females, and further research is needed to examine body representation in male and gender-diverse individuals with AN.

Despite these limitations, the correlation between SO scores in the RHI task and short-term weight change after inpatient treatment highlights the potential utility of body representation assessments in AN treatment. Future larger-scale studies are needed to validate these findings and explore their clinical significance further.

## Conclusion

We found that subsequent weight effects of inpatient treatment of adolescents with AN are associated with the RHI. This has important implications for AN treatment. Although objective measures of body representation are not often used as a treatment, our results suggest that the effectiveness of AN treatment can be partly assessed by body representation. Given the limited sample size, the findings should be interpreted with caution. Future large-scale research is needed to validate the conclusions.

## Supplementary Information

Supplementary Figure S2.

Supplementary Table S1.

## Data Availability

The datasets analysed in this study are available from the corresponding author upon reasonable request.

## Abbreviations

AN: Anorexia nervosa
SO: Sense of ownership
SA: Sense of agency
RHI: Rubber hand illusion
RoHI: Robot hand illusion
HC: Healthy control
IBW: Ideal body weight
BMI: Body mass index
SDS: Standard deviation score
DSRS-C: Birleson Depression Self-Rating Scale for children
SCAS: Spence Child Anxiety Scale
ChEAT26: Children’s Eating Attitudes Test
EDE-Q-J: Eating Disorder Examination Questionnaire Japanese version
