# Supplementary material for: Effects of inpatient treatment of adolescents with anorexia nervosa are associated with body representation: a monocentric pilot study
Source: Sci Rep. 2025 Aug 1;15:28064. doi: 10.1038/s41598-025-13222-w (PMC12317071; doi:10.1038/s41598-025-13222-w)
Supplement: Supplementary file 1 — Supplementary Figure S2. [file 41598_2025_13222_MOESM1_ESM.docx]

**Supplementary Figure S2**

**Spearman's rank correlations between embodiment-related measures and age (HC group).**
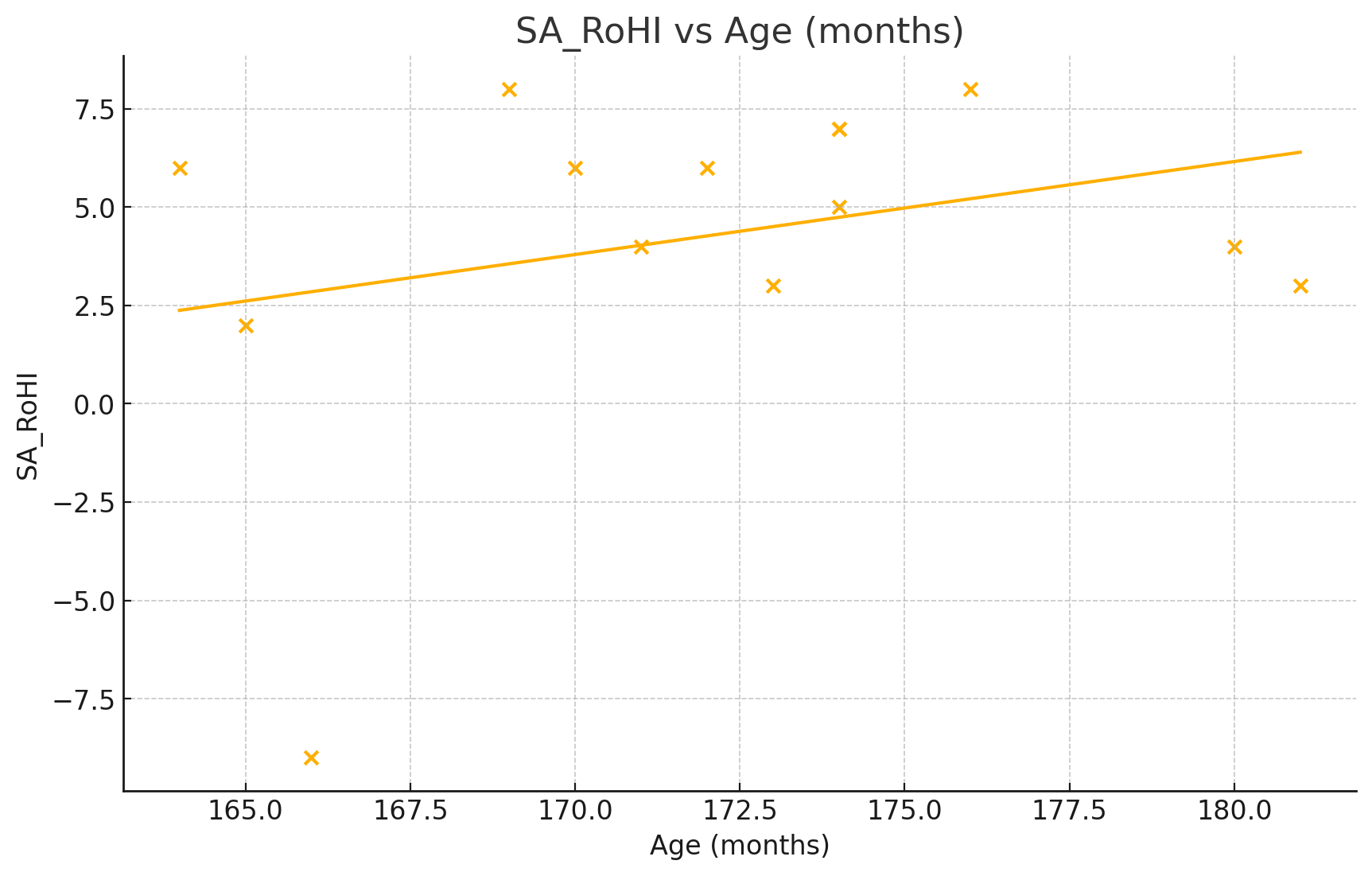

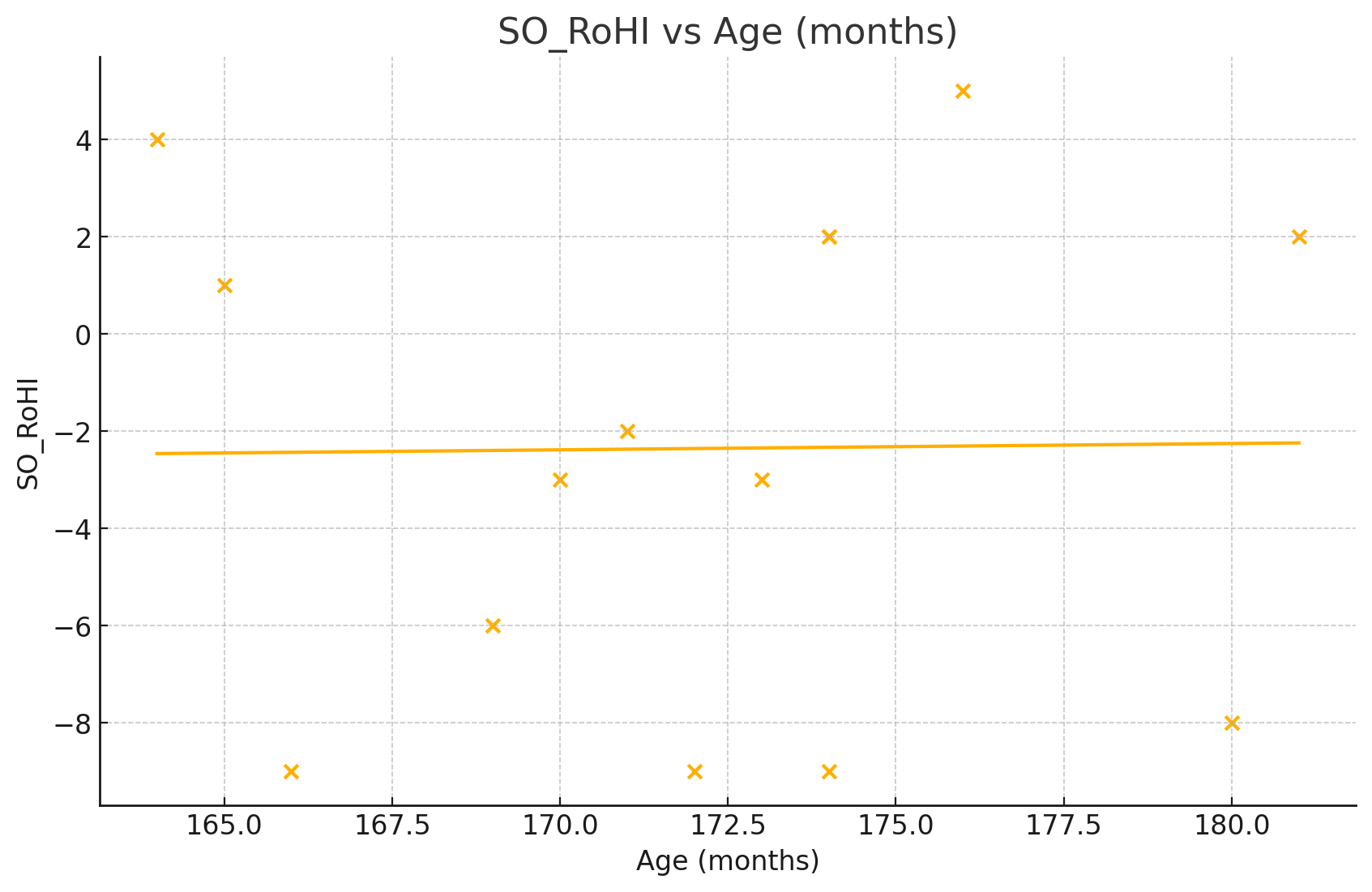

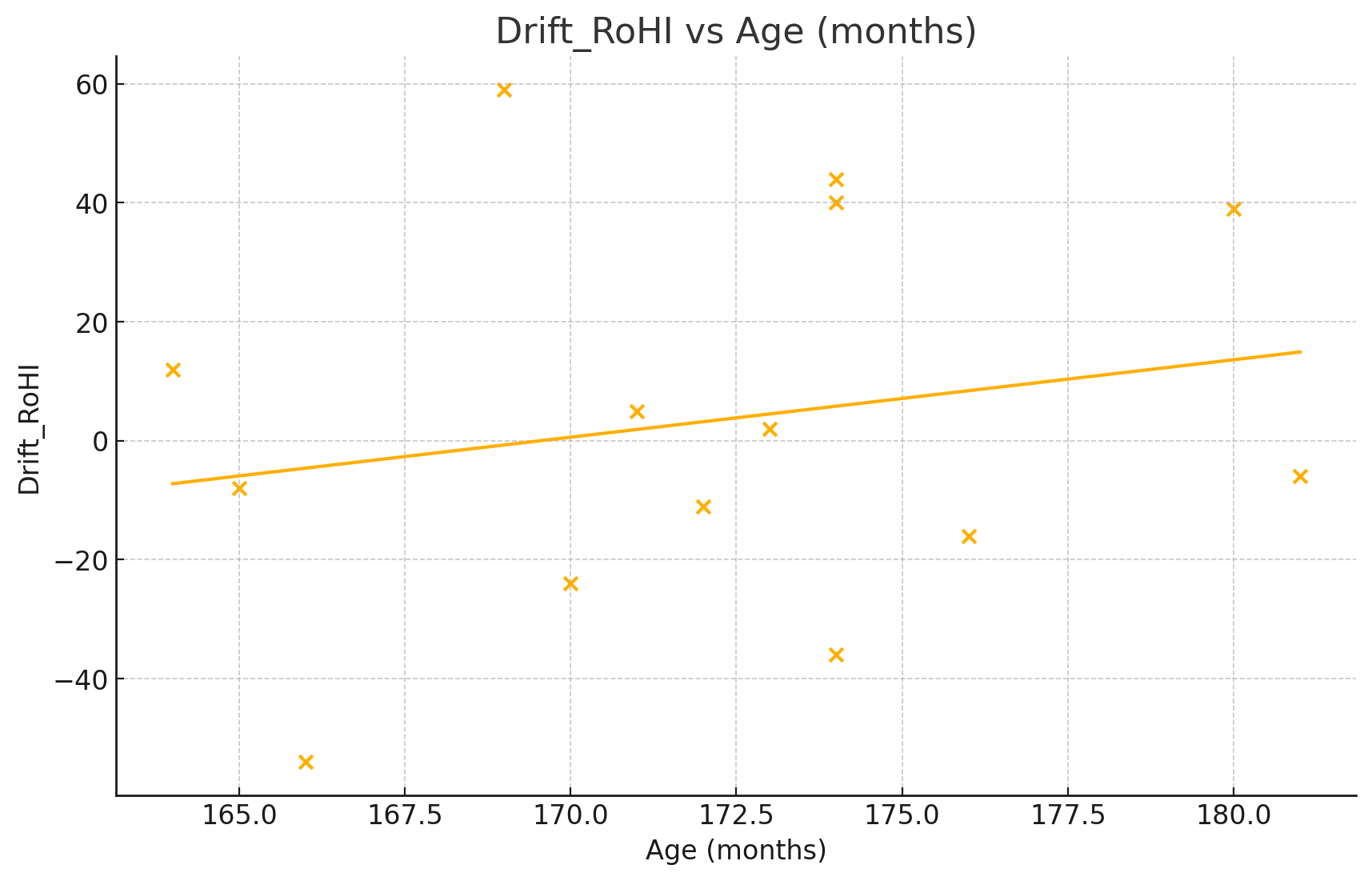

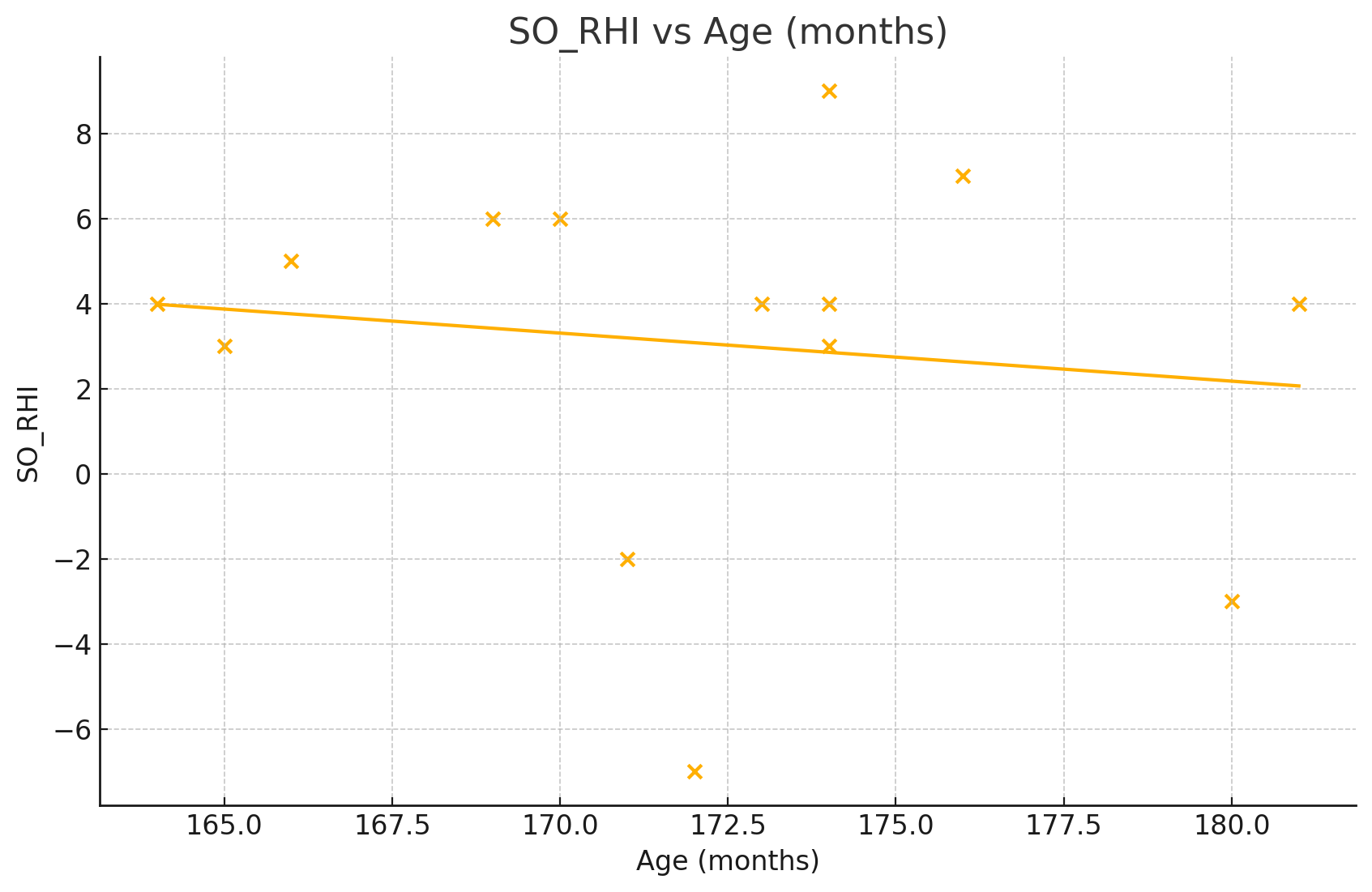

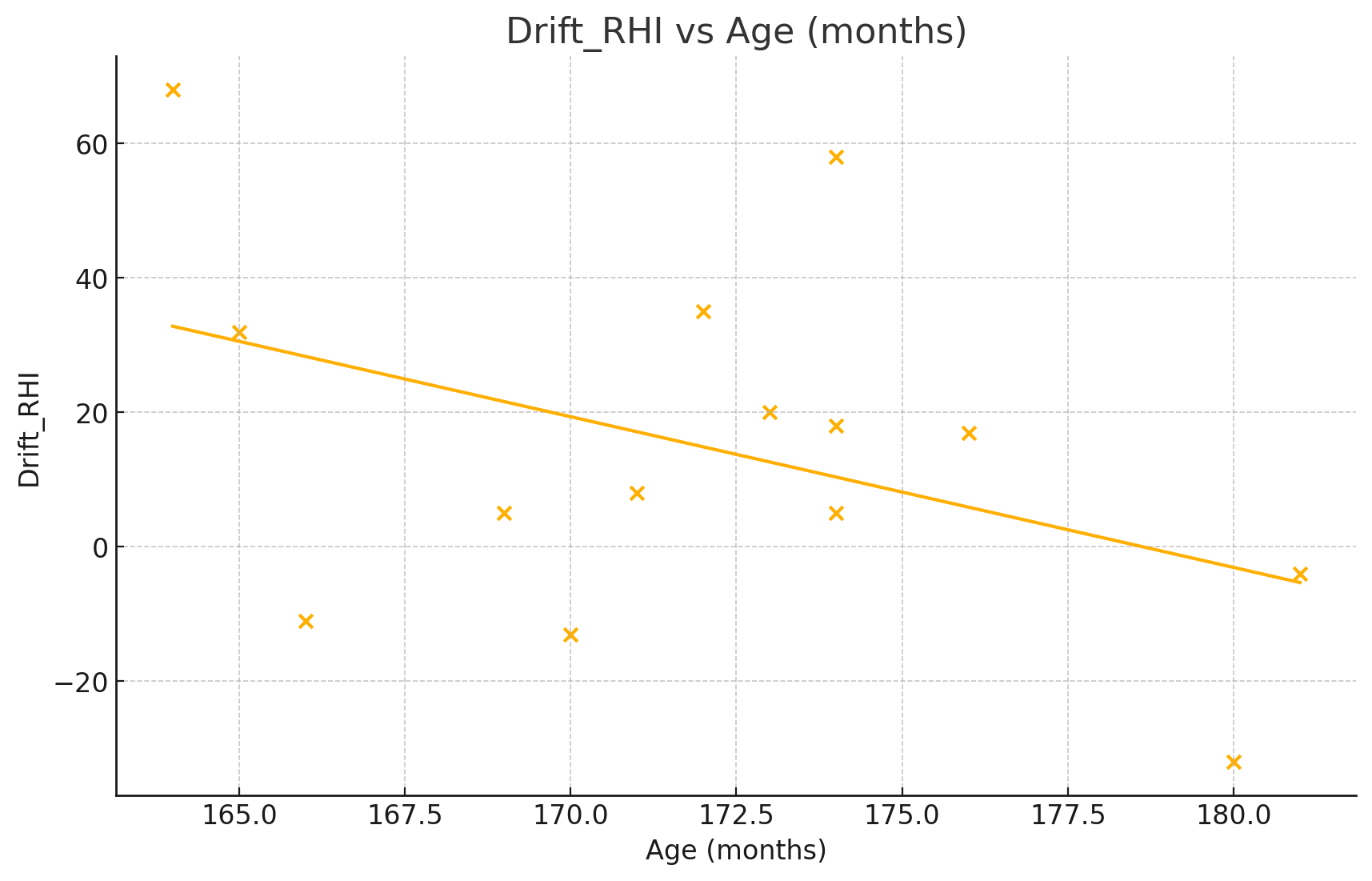


| Measures | ρ (vs age) | *p* |
| --- | --- | --- |
| SA_RoHI | +0.15 | 0.61 |
| SO_RoHI | +0.13 | 0.65 |
| Drift_RoHI | +0.09 | 0.76 |
| SO_RHI | −0.02 | 0.93 |
| Drift_RHI | −0.29 | 0.32 |
