# Supplementary material for: Effects of inpatient treatment of adolescents with anorexia nervosa are associated with body representation: a monocentric pilot study
Source: Sci Rep. 2025 Aug 1;15:28064. doi: 10.1038/s41598-025-13222-w (PMC12317071; doi:10.1038/s41598-025-13222-w)
Supplement: Supplementary file 2 — Supplementary Table S1. [file 41598_2025_13222_MOESM2_ESM.docx]

**Supplementary Table S1**

| Timepoint | measures | ρ (vs %IBW change after discharge) | *p* |
| --- | --- | --- | --- |
| T2 | SO_RHI_T2 | **0.59** | **0.045** |
|  | SA_RoHI_T2 | 0.49 | 0.105 |
|  | Drift_RoHI_T2 | –0.32 | 0.307 |
|  | SO_RoHI_T2 | 0.40 | 0.193 |
|  | Drift_RHI_T2 | –0.23 | 0.480 |
| T1 | SO_RHI_T1 | 0.44 | 0.152 |
|  | SA_RoHI_T1 | –0.23 | 0.480 |
|  | Drift_RoHI_T1 | –0.11 | 0.733 |
|  | SO_RoHI_T1 | –0.40 | 0.198 |
|  | Drift_RHI_T1 | 0.48 | 0.118 |
| T2–T1 | SO_RHI_T2–T1 | 0.12 | 0.721 |
|  | SA_RoHI_T2–T1 | –0.49 | 0.108 |
|  | Drift_RoHI_T2–T1 | –0.31 | 0.307 |
|  | SO_RoHI_T2–T1 | –0.52 | 0.082 |
|  | Drift_RHI_T2–T1 | –0.17 | 0.608 |

**Results of Spearman's rank correlations between %IBW change after discharge and other embodiment-related measures**
